# Childhood motor speech disorders: who to prioritise for genetic testing

**DOI:** 10.1038/s41431-025-01993-9

**Published:** 2026-01-13

**Authors:** Halianna Van Niel, Mariana Lauretta, Emma Baker, Lorraine O’Donnell, Charlotte Boulton, Celia Brenchley, David Coman, Evyenia Michellis, Himanshu Goel, Geoff Thompson, Richard Webster, Georgia Paxton, Zornitza Stark, Ingrid E. Scheffer, Michael S. Hildebrand, David J. Amor, Angela T. Morgan

**Affiliations:** 1 Speech and Language, Genomics Theme, Murdoch Children’s Research Institute, Parkville, Melbourne, VIC Australia; 2 Royal Children’s Hospital, Parkville, Melbourne, VIC Australia; 3 Queensland Children’s Hospital, South Brisbane, QLD Australia; 4 University of Queensland, St. Lucia, Brisbane, QLD Australia; 5 Hunter Genetics, John Hunter Hospital, New Lambton Heights, Newcastle, NSW Australia; 6 Burnside Hospital, Toorak Gardens, Adelaide, SA Australia; 7 Neurology Department, The Children’s Hospital at Westmead, Westmead, NSW Australia; 8 Department of Paediatrics, The University of Melbourne, Melbourne, Australia; 9 Victorian Clinical Genetics Service, Murdoch Children’s Research Institute, Parkville, Melbourne, VIC Australia; 10 Epilepsy Research Centre, Austin Health, Heidelberg, Melbourne, VIC Australia; 11 Genomics Theme, Murdoch Children’s Research Institute, Parkville, Melbourne, VIC Australia; 12 Department of Audiology and Speech Pathology, The University of Melbourne, Melbourne, VIC Australia

**Keywords:** Genetics research, Disease genetics

## Abstract

The aetiology of childhood motor speech disorders of dysarthria and apraxia has been poorly understood. Recent evidence suggests a moderate genetic contribution for these rare and severe speech disorders. To date, however, no studies have examined genetic diagnostic yield for childhood apraxia of speech (CAS) and dysarthria in a clinical setting. Here, we used a clinically accredited genomics pipeline to investigate genetic diagnostic yield and variables predictive of a genetic diagnosis in a tertiary hospital speech clinic. A cohort of 153 children (range 2;7-16;5 years, 42 female) ascertained for motor speech disorder were assessed by a clinical geneticist and speech pathologist and underwent chromosomal microarray, Fragile X and exome sequencing. Odds ratios identified predictors of genetic diagnosis. 44/153 (29%, 15 female) had pathogenic variants (30 de novo), encompassing monogenic conditions (n = 35) and copy number variants (*n* = 9) across 38 distinct disorders. Delayed walking, fine and gross motor disorder, receptive language impairment and/or cognitive impairment, and dysmorphism were associated with a genetic diagnosis. The presence of CAS and dysarthria was more commonly associated with a genetic diagnosis than CAS alone. Autism spectrum disorder was less commonly associated with a genetic diagnosis. No child had a Fragile X diagnosis. The clinical genetic diagnostic yield for motor speech disorders is comparable to epilepsy and cerebral palsy, conditions where genetic testing is routine in most centres, unlike for motor speech disorders. Children with motor speech disorder with co-occurring motor, language and/or learning deficits, should be prioritised for genomic testing.

## Introduction

Speech disorders are a common presenting concern for children referred to paediatricians and primary care physicians [1]. Associated poor language, literacy, educational, employment and social outcomes mean speech disorders come with significant individual, societal and health system burden [2–4].

Up to 5% of children develop common speech disorders, including articulation and phonological impairments [1]. These conditions are highly tractable and typically resolve by 7 years of age, with or without intervention [4, 5]. By contrast, 1 in 1000 children follow a severely disrupted developmental path to intractable motor speech disorders of childhood apraxia of speech (CAS) and/or dysarthria [6]. CAS affects the ability to plan and sequence speech movements, decreasing the precision, consistency and intelligibility of speech [7]. Children with CAS need explicit teaching and practice of new sounds and words, often requiring speech therapy into adolescence or adulthood [8]. A diagnosis of CAS implicates disruption of inferior frontal lobe and basal ganglia pathways, whereas dysarthria is a disorder of the control and execution of neuromuscular movements for speech implicating the ‘final common’ motor pathway or corticobulbar tract [9–11].

A major challenge of managing motor speech disorders has been a lack of aetiological knowledge. Families search for explanatory causes in a long diagnostic journey [12], attending multiple primary care physician, paediatric, neurology, and other specialists appointments, involving serial investigations (e.g. MRI, metabolic testing) with significant costs and psychological stress and without explanatory results [13]. In the past five years, genomic sequencing, performed in a research setting, has shown a substantial genetic contribution to these conditions [14–17], even in the absence of intellectual disability [15, 16], with a diagnostic yield of 30% [3]. This research yield is comparable to that seen in cerebral palsy, epilepsy and intellectual disability, conditions for which genomic clinical testing is routine in many centres [3]. However, clinical genomic testing is not yet the standard of care for motor speech disorders, despite such testing being highly valued by families with lived experience [13].

In this study, we offered clinical genomic testing, consisting of chromosomal microarray, fragile X PCR and exome sequencing, to a clinically ascertained population of children referred to a speech genomics clinic at a paediatric tertiary hospital. We examined diagnostic yield and clinical variables predictive of a genetic diagnosis.

## Materials and methods

### Ethical consent

The study was approved by the Human Research Ethics Committee of The Royal Children’s Hospital, Melbourne, Australia (#37353). Written informed consent was obtained from parents or legal guardians.

### Study design

This prospective single-centre cohort study recruited probands referred to the Genetics of Speech Disorders clinic at the Royal Children’s Hospital in Melbourne, Australia, between August 2020 and December 2023.

### Recruitment

Children were referred for motor speech testing by their treating primary care physician, paediatrician, paediatric sub-specialist (metabolic, genetics, neurology) or speech pathologist. Probands were triaged for eligibility by the clinical nurse co-ordinator with input from the clinical team where indicated (e.g., psychologist to interpret cognitive assessments; clinical geneticist to interpret past genetic reports). Inclusion criteria were: (1) probands aged 2;7 to 17 years with CAS or dysarthria in the absence of known or previously diagnosed intellectual disability, autism spectrum disorder (ASD) level III, or acquired brain injury; and (2) parent, speech pathologist and treating physician reporting the primary presenting feature and clinical concern as motor speech disorder [15, 16].

### Phenotyping

**Fig. 1:**
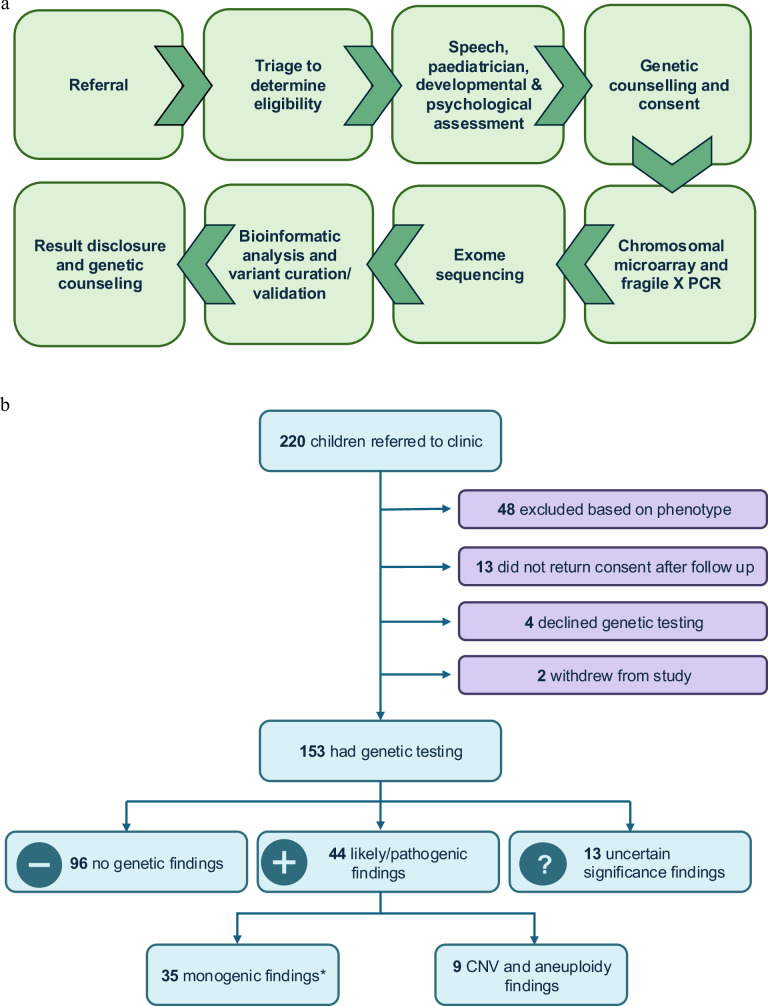
Clinical pipeline. **a** Clinical workflow. PCR Polymerase chain reaction. Referral received by clinical co-ordinator (nurse, LO) at Motor Speech Disorders Clinic. Triage by nurse (LO) and speech pathologist (AM). Proband seen by speech pathologist (AM, ML, CB, AG) to confirm speech motor disorder. Medical work-up by paediatrician and clinical geneticist (DA, dual-qualified). Psychologists (EB, CB) determined IQ status at the further appointment. A genetic counsellor (ML) or a clinical geneticist (DA) performed pre-test genetic counselling and genetic consent. Saliva samples for chromosomal microarray and exome sequencing were collected using paediatric. Oracollect kits from DNAGenotek. Clinically accredited genomic sequencing was performed at the Victorian Clinical Genetics Service, including: initial computational bioinformatics analysis; meeting to prioritise variants for assessment attended by senior medical genomic scientists, clinical geneticists, and genetic counsellors; variant curation as per American College of Medical Genetics and Genomics guidelines. Next, a review of variant and phenotypic data was undertaken by a multidisciplinary speech clinic team, with laboratory scientists, clinical geneticists and genetic counsellors independent to the speech clinic, to reach consensus and ensure genotype concordant with phenotype prior to reporting. Proband attended further clinic appointment (in-person or telehealth) for the return of results with post-test genetic counselling and segregation encouraged where appropriate. **b** Flowchart of recruitment and genetic results. CNV Copy number variant; *Monogenic findings include single nucleotide variants (SNVs) and intragenic deletions.

Probands were prospectively assessed by a speech pathologist (AM, ML, CB); paediatrician and clinical geneticist (DA, dual-qualified); and psychologist (EB, CB) using a protocol outlined below; see clinical pipeline Fig. 1a.

A detailed family history and pedigree were recorded and prospective phenotyping documented medical and developmental history, including the presence of dysmorphism [15, 16] (Table 1, Supplemental Tables 1, 2a, b). All secondary neurodevelopmental outcomes were defined using standardised testing and a professional report for validation, e.g., multi-disciplinary assessment standardised testing for a formal diagnosis of ASD; a physiotherapist confirmed fine or gross motor disorder. Where children had not yet received a formal ASD or attention deficit hyperactivity disorder (ADHD) diagnosis, the paediatrician (DA) recorded the presence of ASD/ADHD features (Table 1; Supplemental Tables 1, 2a, b).

**Table 1:** Phenotype of participants with genetic diagnosis (*n* = 44).

	Sex	Age y;m	Speech diagnosis	Genetic diagnosis	Dys-morphology	IQ	Receptive language	Expressive language	Delayed walking	Gross motor impairment	Fine motor impairment	ASD diagnosis	ADHD diagnosis
**1**	M	5;5	CAS, PDis, Artic	*ADGRL1*	+	Borderline	Severe	Severe	-	-	+	-	-
**2**	M	4;1	CAS	*ANK2*	-		Moderate	Moderate	-	-	-	-	-
**3**	F	5;5	CAS, DYS	*BPTF*	+	Borderline			-	-	+	-	-
**4**	M	4;10	CAS	*CACNA1A*	-		Mild		-	-	+	Features	-
**5**	M	6;10	CAS, DYS	*CACNA1A*	Features	Low average	Severe		+	+	+	-	-
**6**	M	7;3	CAS	*CACNA1A*	-		Average	Severe	+	+	-	Features	Features
**7**	F	3;4	CAS	*CAMK2A*	-	Mild	Average	Low average	+	+	+	-	Features
**8**	M	8;0	CAS, DYS	*CAMTA1*	Features	Low average	Moderate	Severe	+	+	+	-	+
**9**	M	4;8	CAS, PDis	*CUX1*	Features	Borderline	Severe	Mild	-	+	+	+	-
**10**	M	17	DYS	*EBF3*	+	High average	Average	Mild	+	+	+	Features	-
**11**	M	4;3	CAS	*EHMT1*	+	Mild			+	+	-	-	-
**12**	M	6;1	CAS, PDel	*EHMT1*	+	Borderline	Mild	Severe	+	-	+	-	-
**13**	M	6;7	CAS, PDel	*FBXW7*	+	Mild	Severe	Severe	+	+	-	-	-
**14**	M	4;6	CAS, PDel	*FOXP1*	-		Moderate	Severe	+	+	+	Features	-
**15**	F	4;3	CAS, PDis	*GNAI1*	+	Borderline	Mild	Severe	+	+	-	-	-
**16**	F	4;2	CAS	*KCND3*	-	Borderline	Moderate	Moderate	+	+	+	-	-
**17**	M	3;7	CAS, PDis	*KDM5C*	-	Average		Severe	-	-	-	+	Features
**18**	F	5;9	CAS, Stutter, PDel	*NSD1*	+	Borderline	Mild	Severe	+	+	-	-	-
**19**	F	3;7	CAS	*PPP2R5D*	-	Average			+	+	-	-	-
**20**	F	4;6	CAS	*RAF1*	Features		Mild	Severe	-	+	+	Features	-
**21**	F	4;6	CAS	*SCN8A*	Features	Low average	Average	Mild	-	-	+	-	-
**22**	F	10;9	CAS, PDel	*SET*	-	Borderline	Mild	Severe	+	-	-	-	-
**23**	F	3;9	CAS, PDis	*SETBP1*	+	Mild			+	+	+	-	-
**24**	F	14;6	CAS, DYS	*SETBP1*	+	Mild	Mild	Mild	+	+	+	-	-
**25**	M	3;11	CAS	*SETD1A*	+	Low average	Average	Moderate	+	+	+	-	-
**26**	M	4;8	CAS	*SETD2*	Features				-	+	+	-	-
**27**	F	5;2	CAS	*SETD5*	+		Moderate	Severe	+	+	+	-	-
**28**	M	3;5	CAS, PDis	*SLC6A1*	+		Mild	Moderate	-	+	-	-	-
**29**	F	16;5	DYS	*SLC6A8*	Features	Mild	Moderate	Severe	+	+	+	+	-
**30**	F	3;6	CAS, PDis	*SMARCA2*	+	Low average	Low average	Average	-	+	-	-	-
**31**	M	3;9	CAS	*SMARCA2*	+	Mild	Severe	Severe	+	+	-	+	-
**32**	M	7;3	CAS, PDis, Artic	*SPTBN1*	-	Borderline	Severe	Severe	-	+	+	Features	+
**33**	M	13	CAS, PDis	*SRRM2*	+	Borderline			-	+	+	-	-
**34**	M	7;2	CAS	*TAB2*	-	-	Below average	Below average	-	-	+	-	-
**35**	M	4;2	CAS, PDis	*TRIM8*	+	Mild	Severe	Severe	+	+	+	Features	-
**36**	M	5;3	CAS	1q21.1q21.2 dup	-	Average			-	-	-	-	-
**37**	M	9;7	CAS, PDis	15q13.2q13.3 del	-	Borderline	Mild	Severe	-	-	-	-	-
**38**	M	5;1	CAS, PDel	16p12.2 del	Features	Average	Moderate	Average	-	+	+	-	-
**39**	M	5;2	CAS	17q12 dup	-	Average			+	-	+	-	-
**40**	M	4;9	CAS	22q11.21 del	+				+	+	+	-	-
**41**	M	6;6	CAS, Stutter, PDel	22q11.21q11.22 dup	+	Average	Average	Average	-	+	+	-	-
**42**	F	4;1	CAS, DYS	47, XXX	Features	High average	Average		-	+	+	-	-
**43**	M	8;5	CAS, DYS	48, XXYY	+	Low average	Moderate		-	-	-	-	-
**44**	M	6	CAS	48, XYYY	-	Low average	Average	Average	-	-	+	-	-

*F* female, *M* male, y year, *m* month, *CAS* childhood apraxia of speech, *PDis* Phonological disorder, *PDel *Phonological delay, *DYS* Dysarthria, Artic Articulation disorder, + Present, - Absent, *IQ* Full Scale Intelligence Quotient, *blank* not tested, *ASD* Autism spectrum disorder, *ADHD* Attention deficit hyperactivity.

For speech, CAS was confirmed if the American Speech-Language-Hearing Association (ASHA) criteria for CAS were met: (1) inconsistent errors on consonants and vowels in repeated productions of syllables or words, (2) lengthened and disrupted coarticulatory transitions between sounds and syllables, and (3) inappropriate prosody; all relative to age and stage of development [7]. ASHA criteria were operationally defined and rated from: phonetic transcriptions of 55 single-word speech tests designed to elicit all sounds of English, a test of speech consistency (children repeat 15/55 stimuli words twice) and a 5 min conversational speech sample as described previously [15, 16, 18]. Dysarthria was diagnosed as an oral tone or coordination disturbance, examined by a paediatrician and speech pathologist and speech dysarthric features were identified during the conversation sample, using the Mayo Clinic Dysarthria rating scale [15, 16, 18, 19]. Receptive and expressive language was assessed where time and resources were available [20–23] (Table 1, Supplemental Tables 1, 2a, b). Cognition was assessed by a psychologist using standardised testing [24–26].

### Genetic and genomic analysis

A genetic counsellor (ML) and/or clinical geneticist (DA) provided pre and post-genetic counselling. Chromosomal microarray analysis (CMA, Illumina GSA, San Diego, USA) and Fragile X PCR sizing were performed on saliva or blood DNA for all probands prior to exome sequencing (ES). Saliva samples were collected using paediatric Oracollect kits from DNAGenotek. For ES, genomic DNA was isolated from saliva samples with trio testing (probands together with parents) performed where possible. ES was performed by Victorian Clinical Genetics Services (VCGS), Melbourne, see Supplemental Fig. 1 for an overview of the analysis pipeline. Coding regions were enriched using TWIST exome capture (TWIST Bioscience). Sequencing was performed on Illumina sequencers to an average target depth of 80×. Data were analysed using an in-house validated version of Cpipe [27], followed by variant filtering and analysis using Alissa Interpret analysis software (Agilent). Data were analysed for variants linked to disorders relevant to the proband phenotype following a tiered approach. Initially, variants in genes associated with intellectual disability were evaluated (https://panelapp-aus.org/panels/250/). Analysis was then expanded to a virtual panel of ~6000 genes known to cause Mendelian disorders (https://panelapp-aus.org/panels/137/). Variants were classified according to the current American College of Medical Genetics and Genomics (ACMG) guidelines for variant interpretation [28].

### Statistical analysis

We report the proportion and type of pathogenic variants causative for motor speech disorder from CMA, Fragile X and ES testing. For variables predictive of a genetic diagnosis, baseline clinical variables are recorded as frequencies (*n*, %) and medians (range), as dictated by data type. Odds ratios have been used to identify variables associated with a genetic diagnostic result. The variables of ASD, ADHD, dysmorphism, intelligence quotient (IQ) and receptive and expressive language were all dichotomised for the odds ratio analysis.

## Results

### Genetic diagnostic yield and genomic findings

172 probands met the inclusion criteria, of whom 153 (88.9%; 111 males, 42 females) completed genetic testing (153 CMA, 143 ES), at an average age of 5 years 8 months (range 2 years 7 months to 16 years 5 months). Two probands withdrew from the study and 4 declined genetic testing. Despite three reminders to do so, the remaining 13 either did not respond to the genetic testing appointment invitation, did not send back their consent form, or did not return their saliva sample (Fig. 1b).

Overall, 44/153 (29%) probands received a genetic diagnosis (15 females; median 5 years 1 month; Tables 1, 2). 30 findings were confirmed de novo and 9 inherited (5 maternal; 4 paternal). Inheritance status could not be confirmed for 5 pathogenic variants due to missing parental DNA (Table 2, Supplemental Fig. 1a). 9 were aneuploidies or copy number variants (CNVs) (all >50 kb) detected on CMA: 15q13.2q13.3 del, 22q11.21 del, 22q11.21q11.22 dup, 1q21.1q21.2 dup, 16p12.2 del, 17q12, 47 dup, 47,XXX, 48,XXYY, 48,XYYY. There were 35 monogenic diagnoses, including single-nucleotide variants (SNVs) and intragenic deletions (< 50 kb). 34/35 were identified from ES: *ADGRL1*, *ANK2*, *BPTF*, *CACNA1A* (*n* = 3), *CAMK2A*, *CAMTA1*, *EBF3*, *EHMT1* (*n* = 2), *FBXW7*, *FOXP1*, *GNAI1*, *KCND3, KDM5C*, *NSD1*, *PPP2R5D*, *RAF1*, *SCN8A*, *SET*, *SETBP1* (*n* = 2), *SETD1A, SETD2*, *SETD5*, *SLC6A1*, *SLC6A8, SMARCA2* (*n* = 2), *SPTBN1, SRRM2, TAB2*, *TRIM8*. 1/35 was identified from CMA*:*

**Table 2:** **(a)** Monogenic findings (including single nucleotide variations and intragenic deletions), *n* = 35; (**b**) Copy number variants (CNVs) and aneuploidies, *n* = 9 (Genetic diagnoses *n* = 44).

(a)						
ID	Gene	Variant (GRCh38/hg38)	Chr	Inheritance	ACMG Class	Type
1	*ADGRL1*	g.(?_14150873)_(14152912_?)del	19	Maternal	4	Intragenic deletion exon 19-23
2	*ANK2*	c.9539_9545delinsTGGATGATGAG; p.(Asp3180Valfs*7)	4	Paternal	5	Frameshift
3	*BPTF*	c.3157_3158delAA; p.(Lys1053Glufs*12)	17	De novo	5	Frameshift
4	*CACNA1A*	c.3829 C > T; p.(Arg1277*)	19	Paternal	5	Nonsense
5	*CACNA1A*	c.492 C > G; p.(Tyr164*)	19	Maternal	5	Nonsense
6	*CACNA1A*	c.592 C > T; p.(Arg198*)	19	Maternal	5	Nonsense
7	*CAMK2A*	c.635 C > T; p.(Pro212Leu)	5	De novo	5	Missense
8	*CAMTA1*	c.2072_2075del; p.(Thr691Argfs*35)	1	De novo	5	Frameshift
9	*CUX1*	g.102070411_102114170del	7	Unknown	4	Intragenic deletion exon 4-7
10	*EBF3*	c.708_710delCAA; p.(Asn237del)	10	De novo	5	Deletion
11	*EHMT1*	c.3229 C > T; p.Gln1077*	9	Unknown	5	Nonsense
12	*EHMT1*	c.2842 C > T; p.(Arg948Trp)	9	De novo	5	Missense
13	*FBXW7*	c.1919delG; p.(Ser640Thrfs*7)	4	Unknown	4	Frameshift
14	*FOXP1*	c.1426 C > T; p.(Gln476*)	3	Unknown	4	Nonsense
15	*GNAI1*	c.518 A > T; p.(Asp173Val)	7	De novo	4	Missense
16	*KCND3*	c.983 T > G; p.(Leu328Arg)	1	De novo	4	Missense
17	*KDM5C*	c.1178 C > T; p.(Thr393Ile)	X	De novo	4	Missense
18	*NSD1*	c.4972_4974delTTG; p.(Leu1658del)	5	De novo	4	Deletion
19	*PPP2R5D*	c.751 G > T; p.(Asp251Tyr)	6	De novo	5	Missense
20	*RAF1*	c.1423 T > C; p.(Phe475Leu)	3	De novo	5	Missense
21	*SCN8A*	c.417 G > A; p.(Met139Ile)	12	De novo	5	Missense
22	*SET*	c.103_104del; p.(Ile35*)	9	De novo	4	Nonsense
23	*SETBP1*	c.623del; p.(Pro208Glnfs*135)	18	De novo	5	Frameshift
24	*SETBP1*	c.2087dupC; p.(Glu697Argfs*10)	18	De novo	5	Frameshift
25	*SETD1A*	c.273del; p.(Pro92Hisfs*2)	16	De novo	5	Frameshift
26	*SETD2*	c.6284dup; p.(Asp2096Argfs*2)	3	De novo	5	Frameshift
27	*SETD5*	c.2347-7 A > G	3	De novo	5	Splicing
28	*SLC6A1*	c.1097_1098delinsCT; p.(Leu366Pro)	3	De novo	4	Missense
29	*SLC6A8*	c.257 G > A; p.(Gly86Asp)	X	De novo	4	Missense
30	*SMARCA2*	c.3484 C > T; p.(Arg1162Cys)	9	De novo	5	Missense
31	*SMARCA2*	c.2870 A > G; p.(Gln957Arg)	9	De novo	4	Missense
32	*SPTBN1*	c.1429 C > T; p.(Arg477Cys)	2	De novo	4	Missense
33	*SRRM2*	g.2707830_2757509del	16	De novo	5	Intragenic deletion of exon 1,2 and part of 3
34	*TAB2*	c.363_364insGTTA; p.(Phe122Valfs*18)	6	Maternal	5	Frameshift
35	*TRIM8*	c.1357 C > T; p.(Gln453*)	10	De novo	5	Nonsense

(b)			
ID	Genetic finding	Molecular karyotype (GRCh38/hg38)	Inheritance
36	1q21.1q21.2 dup	1q21.1-21.2(chr1:146891138-148353326)x3	Paternal
37	15q13.2q13.3 del	15q13.2-13.3(chr15:30648301-32222899)x1	Unknown
38	16p12.2 del	16p12.2(chr16:21945136-22403142)x1	Paternal
39	17q12 dup	17q12(chr17:36459737-37889808)x3	Maternal
40	22q11.21 del	22q11.21(chr22:18890274-20324123)x1	De novo
41	22q11.21q11.22 dup	22q11.21-11.22(chr22:21450614-22457038)x3	De novo
42	47,XXX	(chrX)x3	De novo
43	48, XXYY	(chrX)x2, (chrY)x2	De novo
44	48, XYYY	(chrX)x1, (chrY)x3	De novo

*CUX1* (Tables 1, 2). Variant types for SNVs (*n* = 32) included missense (*n* = 13), frameshift (*n* = 9), nonsense (*n* = 7), in-frame deletions (*n* = 2), and splicing (*n* = 1) (Table 2). All variants were deposited in ClinVar (https://www.ncbi.nlm.nih.gov/clinvar/; accession numbers SCV002557839.2- SCV005399598.1).

13 candidate variants of uncertain significance (VUS) were identified in probands without a diagnosis; comprising SNVs in the following genes: *AP1G, BCORL1, BRPF1, CRY, DPF2, FGF13, IQSEC2, USP9X* (*n* = 2*)*; and the following CNVs: 14q12 del (de novo), 7p22.3 del (maternal), 3p14.2 del (de novo), and 2q37.2q37.3 del (maternal) (Supplemental Table 3). Two additional VUSs were identified in solved probands ID8 *FOXP2*, and ID5 *TRRAP* [28].

All (likely) pathogenic variants identified were in genes previously associated with syndromic or non-syndromic intellectual disability, epilepsy, ASD and cerebral palsy, Supplemental Table 4. No child received a diagnosis of Fragile X syndrome or Fragile X-associated disorder/pre-mutation carrier status.

### Phenotyping: variables predictive of a genetic diagnosis

**Fig. 2:**
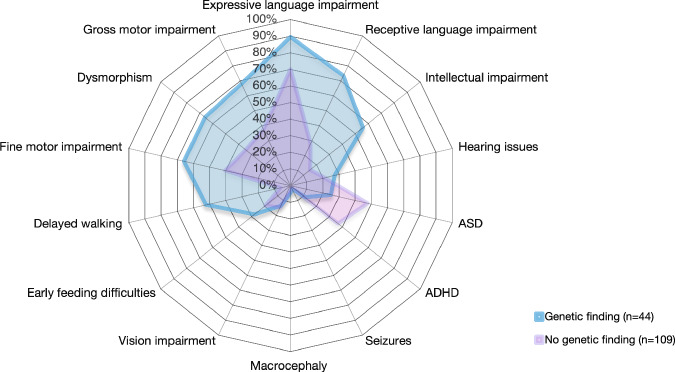
Radar chart of phenotypic overlap for participants with and without clinical genetic diagnoses. ADHD ADHD diagnosis or ADHD features, ASD ASD diagnosis or ASD features, Dysmorphism Dysmorphic or dysmorphic features, Intellectual impairment: borderline ID or mild ID; Expressive language impairment: mildly impaired, moderately impaired, severely impaired, minimally verbal or could not be assessed due to severity; Receptive language impairment: mildly impaired, moderately impaired or severely impaired.

Phenotypes of 44 probands with a genetic diagnosis, and 109 without a genetic diagnosis, are presented in Fig. 2 and Supplemental Tables 1, 2, 5. Compared to those without a genetic diagnosis, a higher proportion of children with genetic diagnoses had dysarthria (with and without CAS; 18.2% vs 8.3%), gross motor delays (68.2% vs. 37.6%), fine motor delays (65.9% vs. 40.4%), or a delayed age of walking (52.3% vs. 6.4%), dysmorphism (45.5% vs. 5.5%) and/or borderline or mild intellectual disability (55.9% vs. 15.2%); and a lower proportion had confirmed ASD (9.1% vs 18.3%). No participant with a genetic diagnosis had seizures or a diagnosis of epilepsy, although participant 6 had staring spells with a normal EEG. Of those without genetic diagnoses, only two had seizures; participant 147 had absence seizures; participant 71 had a history of febrile seizures.

No potential environmental factors contributing to CAS were identified. No probands had a history of congenital infection, teratogen exposure, birth injury, severe prematurity or acquired brain injury. Six probands had a history of moderate to late prematurity (32–26 weeks) and none of these had a genetic diagnosis.

Consistent with these descriptive findings, the odds of having a genetic diagnosis were highest for individuals with delayed age of walking (OR:15.96, 95% CI: 6.34, 44.86), receptive language impairment (OR: 7.14, 95% CI: 2.96, 18.62), borderline or mildly impaired intellect (OR: 7.07, 95% CI: 2.89, 18.18), dysmorphism (present/features) (OR: 5.59, 95% CI: 2.66, 12.19), expressive language impairment (OR: 3.62, 95% CI: 1.28, 13.04), gross motor delay/disorder (OR: 3.55, 95% CI: 1.72, 7.65); and fine motor delay/disorder (OR: 2.86, 95% CI: 1.39, 6.06), respectively (Supplemental Table 5). The odds of having a genetic diagnosis were lowest for children with confirmed diagnoses of ADHD or ASD. Wide confidence intervals for the OR reflect the relatively small sample size for children with a genetic diagnosis.

## Discussion

We identified a specific genetic diagnosis in 29% of children referred to a motor speech disorders clinic at a paediatric tertiary hospital. This yield replicates research findings in a clinical setting for the first time. Previous prospective research-based cohorts ascertained for a primary motor speech diagnosis have identified genetic diagnoses in 42%, 33% and 26% respectively [14–16], but may have been subject to ascertainment bias. Importantly, this diagnostic yield is comparable to that of genomic testing in other neurodevelopmental conditions, such as epilepsy, cerebral palsy and intellectual disability, for which genomic testing is increasingly routine [29–32].

Mendelian diagnoses were present in 23% (35/153) of the cohort. We implicate 15 Mendelian disorder genes as novel explanations for CAS and dysarthria: *ADGRL1*, *ANK2, BPTF, CAMK2A, CUX1, FBXW7, KCND3, NSD1, RAF1, SETD2, SLC6A8, SPTBN1, SRRM2, TAB2, TRIM8)*. We also confirm involvement of *CACNA1A, CAMTA1*, *EBF3 EHMT1, GNAI1, KDM5C, PPP2R5D, SCN8A, SET, SETBP1, SETD1A, SETD5, SLC6A1, SMARCA2* and *FOXP1*, now recurrent in unrelated cases across cohorts ascertained for CAS and dysarthria [14–17, 33]. Past work led to the development of the first publicly available clinical speech panel in July 2024, which we have now expanded to include newly implicated genes with sufficient evidence for inclusion (https://panelapp-aus.org/panels/4290/). We highlight that two of our implicated genes are associated with multiple speech disorder phenotypes: *SPTBN1* in CAS, stuttering [34] and speech delay [35]; and *SETD1A* in CAS and speech delay [35].

Although we excluded children with a pre-existing diagnosis of intellectual disability, our subsequent assessment indicated that 9.7% had mild and 17.7% had borderline intellectual disability. Probands with a genetic diagnosis were more likely to have cognitive impairment compared to those without a genetic diagnosis. This result was not unexpected, given that all genes associated with CAS to date are also associated with intellectual disability [3]. Of those children with a genetic diagnosis and normal IQ, most had an IQ at the lower end of the normal range; although, there were some exceptions: an IQ above 100 was recorded in female participant 47 with 48,XXX (Trisomy X), male participant 10 with an *EBF3* variant, and male participant 38 with 16p12.2 deletion. Children with a genetic diagnosis were also more likely to have impairments in motor domains, with delays in walking and fine and gross motor function commonly reported (52.3%, 65.9%, 68.2%, respectively). Overall, these data support the hypothesis that in monogenic forms of CAS, the speech phenotype is part of a broader neurodevelopmental impairment. We also noted that children with CAS and co-occurring dysarthria were more likely to have a genetic diagnosis than children with CAS alone. This is the first evidence of this observation to our knowledge, however requires independent replication.

It is notable that children with a genetic diagnosis ascertained via CAS and/or dysarthria in our cohort were typically towards the milder end of the reported severity spectrum for their disorder. This is likely explained by ascertainment biases favouring (1) severe cases in the published literature, and (2) milder cases in our CAS cohort, also seen in our past studies [15, 16]. For example, we identified *CACNA1A* variants in three unrelated probands in the absence of epilepsy or intellectual disability [36]. By extension from these data, milder forms of monogenic neurodevelopmental disorders are likely to be more common than currently recognised, and a case can be made for expanding the application of genomic sequencing to children (and adults) with mild and borderline ID.

In the 71% of our cohort without a specific genetic diagnosis, a different phenotypic profile emerged. A significant majority of this group had average FSIQ and expected age of walking, and nearly half (47.7%) had either features of ASD or a formal ASD diagnosis. The genetic basis of these undiagnosed cases remains unclear, but the clinical overlap with ASD implicates polygenic inheritance [37, 38]. Based on these observations, we hypothesise that the genetic architecture of CAS comprises at least two distinct causal pathways: (1) monogenic CAS, characterised by variable cognitive impairment, motor impairment and syndromic features, and (2) polygenic CAS, in which cognition and motor skills are relatively spared, but ASD features are more common.

The association between CAS abnormalities on CMA has yet to be fully characterised. Only 4% of our cohort had a copy number variant. All six CNVs in our study are recognized recurrent CNVs with incomplete penetrance, and each was detected in only one patient. An additional 2% of patients had sex chromosome aneuploidies, including two patients with four sex chromosomes (XXYY and XYYY). The association between sex chromosome trisomies and quadrisomies has been previously recognized [39]. An overall yield of 6% for CMA in CAS is lower than reported previously in an Italian CAS cohort (13%) [17] and for intellectual disability, but comparable to the yield in some other neurodevelopmental phenotypes, such as autism and epilepsy, and microarray remains an important front-line test for motor speech disorders. By contrast, we found no cases of fragile X (neither premutation nor full mutation), in line with past prospective studies in the field, suggesting that this test may be less useful in children with CAS [14–17]. Past retrospective chart review studies of large cohorts reporting on genotype-CAS phenotype associations have also failed to identify any association with fragile X [33, 40].

We found no significant evidence for environmental factors contributing to CAS, including pregnancy-related and neonatal complications. Moderate to late prematurity (32-36 weeks) was reported in 5.5% of patients without a genetic diagnosis, comparable to the general population. Prematurity was not seen in any participants with a genetic diagnosis. Our data are in line with past studies showing no evidence of CAS or dysarthria diagnoses in children born pre-term [41].

The range of genetic diagnoses in our cohort underscores the role of chromatin modifiers and transcriptional regulators (BPTF, CAMTA1, CUX1, EBF3, EHMT1, FOXP1, KDM5C, NSD1, SET, SETD1A, SETD2, SETD5, SMARCA2, SETBP1, SRRM2) in speech disorder pathogenicity, consistent with our prior reports [3]. We highlight the commonality of genes encoding SET domain proteins (SETD1A, SETD2, SETD5, NSD1, EHMT1), as these are part of a methyltransferase superfamily integral in post-translational protein regulation with a well-established role in neurodevelopmental disorders [15, 16]. Other protein classes enriched for pathogenic variants were ion channels and solute carriers (ANK2, SCN8A, KCND3, CACNA1A, SLC6A1, SLC6A8) and cell signalling pathway members (FBXW7, PPP2R5D, TAB2, RAF1, SET).

Whilst further replication and verification of results is warranted, given the modest size of our cohort, motor speech disorders are extremely rare (1 in 1000) when compared to other neurodevelopmental conditions (1 in 100 for intellectual disability, autism; 4-10 per 1000 for epilepsy) and greater collaboration will be necessary to build sizeable cohorts for independent replication.

While this research focused on a clinical diagnostic setting, future gene discovery research outside of a clinical pipeline remains important to explore novel variants associated with CAS, with functional characterisation.

## Conclusion

Our findings confirm a diagnostic yield for motor speech disorder that is comparable to other neurodevelopmental conditions. We have identified predictors of genetic diagnosis that can be used to guide advances in clinical practice and health services for motor speech disorder. We suggest prioritisation of clinical genomic testing for children with a primary motor speech disorder on a background of delayed walking, persistent fine and gross motor delays/disorder, receptive language impairment, dysmorphism, and/or borderline IQ. The presence of ASD appears to be less commonly associated with a genetic diagnosis. Motor speech conditions are distinctive, socially debilitating disorders, and clinical genomic testing should be warranted to end the diagnostic odyssey and position them for precision therapies.

## Supplementary information

Supplemental Figure 1

Supplemental Table 1

Supplemental Table 2a

Supplemental Table 2b

Supplemental Table 3

Supplemental Table 4

Supplemental Table 5

## Data Availability

The datasets are included in this study and in supplementary information files or are available from the corresponding author on reasonable. Variant data is available in ClinVar (https://www.ncbi.nlm.nih.gov/clinvar/).
