# Supplementary material for: Childhood motor speech disorders: who to prioritise for genetic testing
Source: Eur J Hum Genet. 2026 Jan 13;34(5):639–48. doi: 10.1038/s41431-025-01993-9 (PMC13171898; doi:10.1038/s41431-025-01993-9)

Supplementary Figure 1. Extended methods figure

1. Exome sequencing cohort, b) bioinformatic analysis, c) variant curation/validation


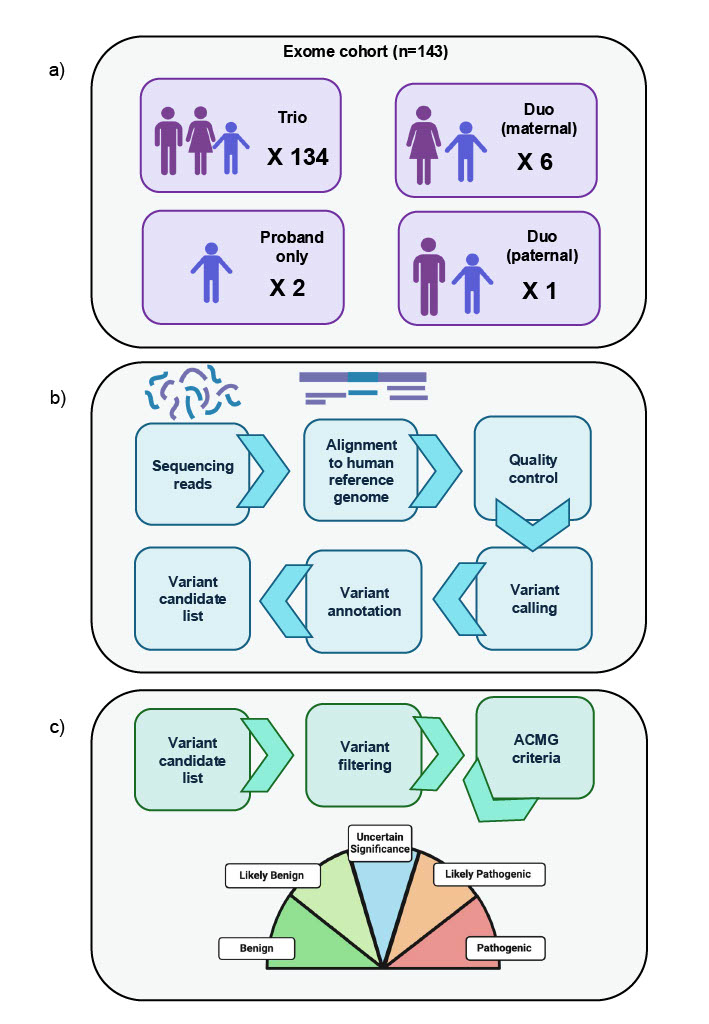

Supplement: Supplementary file 1 — Supplemental Figure 1 [file 41431_2025_1993_MOESM1_ESM.docx]
