# Supplementary material for: Childhood motor speech disorders: who to prioritise for genetic testing
Source: Eur J Hum Genet. 2026 Jan 13;34(5):639–48. doi: 10.1038/s41431-025-01993-9 (PMC13171898; doi:10.1038/s41431-025-01993-9)
Supplement: Supplementary file 2 — Supplemental Table 1 [file 41431_2025_1993_MOESM2_ESM.docx]

| ID | Sex | Age y;m | Speech diagnosis | Dys-morphology | IQ | Receptive language  impairment | Expressive language  impairment | Delayed walking | Gross motor impairment | Fine motor impairment | ASD diagnosis | ADHD diagnosis |
| --- | --- | --- | --- | --- | --- | --- | --- | --- | --- | --- | --- | --- |
|  | M | 2;7 | CAS | - | Average | Moderate | Severe | - | - | - | Features | - |
|  | M | 3;1 | CAS | - | Low average |  |  | - | - | + | Features | - |
|  | M | 3;1 | CAS | - | Borderline |  |  | - | + | + | Features | - |
|  | M | 3;2 | CAS | - | High average |  |  | - | + | + | + | + |
|  | M | 3;2 | CAS, Stutter | - | Low average | Average | Severe | - | + | + | Features | - |
|  | M | 3;2 | CAS | Features |  |  |  | - | - | - | - | - |
|  | M | 3;3 | CAS, PDis | - | Average | Average | Severe | - | - | - | - | - |
|  | F | 3;4 | CAS | Features | Average | Mild | Average | - | - | + | - | - |
|  | F | 3;4 | CAS | Features | High average | Above average | Average | - | + | + | + | - |
|  | M | 3;4 | CAS | Features |  | Mild | Severe | - | - | + | - | - |
|  | M | 3;5 | CAS | - | Low average | Average | Average | - | - | - | + | + |
|  | F | 3;5 | CAS, DYS | Features | Very superior |  |  | - | - | + | Features | Features |
|  | M | 3;6 | CAS, PDel | - |  | Average | Average | - | + | - | - | - |
|  | M | 3;6 | CAS | - |  | Average | Moderate | - | - | - | - | - |
|  | M | 3;6 | CAS | Features |  | Average | Below average | - | + | + | - | - |
|  | M | 3;7 | CAS | - | Average |  |  | - | - | - | Features | + |
|  | M | 3;7 | CAS | - | Average | Average | Severe | - | - | - | + | - |
|  | M | 3;7 | CAS | - |  |  |  | - | + | + | Features | - |
|  | F | 3;8 | CAS | Features | Low average | Average |  | + | + | - | - | - |
|  | M | 3;8 | CAS | - | Average |  |  | - | - | - | - | - |
|  | M | 3;11 | CAS | Features | Borderline |  |  | - | + | - | Features | - |
|  | M | 3;11 | CAS, PDel | - |  | Average |  | - | - | - | - | - |
|  | M | 4 | CAS | - | Average | Average | Severe | - | - | - | Features | Features |
|  | F | 4 | CAS | Features | Low average |  |  | - | + | - | Features | Features |
|  | M | 4 | CAS | - | High average | Average | Severe | - | - | - | - | - |
|  | M | 4;0 | CAS | - | Mild | Average | Severe | - | - | + | Features | Features |
|  | M | 4;0 | CAS | Features | Average | Average | Moderate | - | - | - | - | - |
|  | M | 4;1 | CAS, PDis | - | Average | Average | Severe | - | - | - | - | - |
|  | M | 4;2 | CAS, PDis | - | High average | Average |  | - | + | + | Features | - |
|  | M | 4;2 | CAS | - |  |  |  | - | - | - | Features | - |
|  | F | 4;3 | CAS | - | Average | Average | Severe | - | - | - | Features | - |
|  | M | 4;3 | CAS | - | High average | Average | Average | - | - | - | - | + |
|  | F | 4;4 | CAS, PDel | - | Superior |  |  | - | - | - | - | - |
|  | M | 4;4 | CAS | - | Average |  |  | - | - | - | - | - |
|  | F | 4;4 | CAS, DYS | - | Low average |  |  | - | + | + | - | - |
|  | M | 4;4 | CAS | - |  |  |  | - | + | + | Features | - |
|  | M | 4;4 | CAS | - | Low average |  | Severe | + | + | - | - | - |
|  | M | 4;4 | CAS, PDis | Features | High average |  |  | - | - | - | - | - |
|  | M | 4;5 | CAS | - | High average | Average | Average | - | - | + | Features | Features |
|  | M | 4;6 | CAS, PDel | - |  | Average | Mild | - | - | - | Features | Features |
|  | F | 4;7 | CAS,  DNS | Features |  | Average | Average | - | + | + | Features | - |
|  | M | 4;8 | CAS, PDis | - | Average | Average | Mild | - | - | - | Features | - |
|  | F | 4;8 | CAS, Artic | - |  |  |  | + | - | - | Features | - |
|  | M | 4;8 | CAS | - | Average | Above average | Average | - | - | - | - | - |
|  | M | 4;8 | CAS | - |  | Average | Average | - | - | - | Features | - |
|  | F | 4;9 | CAS | Features | Low average | Mild | Mild | + | - | - | - | - |
|  | M | 4;9 | CAS | + | Borderline | Mild | Severe | - | - | + | Features | Features |
|  | M | 4;9 | CAS | - | Average | Average |  | - | + | + | - | - |
|  | M | 4;9 | CAS, PDel | - |  |  |  | - | - | - | - | - |
|  | F | 4;9 | CAS | - |  | Average |  | - | - | + | + | - |
|  | M | 4;10 | CAS | - | Average | Above average | Average | - | + | + | - | - |
|  | F | 5 | CAS, PDis | Features | Borderline |  |  | - | - | - | + | Features |
|  | F | 5 | CAS, PDis | - |  | Average | Average | + | + | - | - | - |
|  | M | 5;0 | CAS, PDis | - | Average | Average | Mild | - | - | - | - | - |
|  | F | 5;1 | CAS | - | Average | Average |  | - | - | - | - | - |
|  | F | 5;1 | CAS, PDel | - |  |  |  | + | + | - | + | - |
|  | M | 5;1 | CAS | - | Low average | Above average | Average | - | - | - | - | + |
|  | M | 5;2 | CAS, PDis | Features | Average | Average | Severe | - | - | + | + | - |
|  | F | 5;2 | CAS, PDel | Features | Low average |  |  | - | - | - | - | - |
|  | F | 5;3 | CAS, PDis | - | Superior | Average | Average | - | + | - | - | - |
|  | M | 5;4 | CAS, PDel | - | Low average | Moderate | Severe | - | - | - | - | - |
|  | M | 5;4 | CAS | - | Average | Average | Average | - | + | - | - | + |
|  | M | 5;5 | CAS, DYS | + | Low average |  |  | - | - | + | - | - |
|  | M | 5;5 | CAS, PDel | - |  |  |  | - | + | - | Features | - |
|  | M | 5;6 | CAS | - | Average | Average | Moderate | - | - | - | Features | Features |
|  | M | 5;6 | CAS, PDis | + | High average | Average | Average | - | - | + | Features | + |
|  | F | 5;6 | CAS, DYS, PDel | Features |  | Mild | Severe | - | + | - | - | - |
|  | M | 5;6 | CAS, PDel | - |  |  |  | - | - | - | - | Features |
|  | M | 5;7 | CAS, PDis | - |  |  |  | - | - | - | - | Features |
|  | M | 5;9 | CAS, PDis | - | Low average | Mild | Severe | - | + | + | - | + |
|  | M | 5;9 | CAS, PDel | Features | Low average | Average | Average | + | + | + | Features | - |
|  | M | 5;9 | CAS | - | Average | Average | Average | - | - | - | - | - |
|  | M | 5;10 | CAS, PDel | - |  | Average | Average | - | + | + | - | - |
|  | M | 5;11 | CAS, DNS | - |  | Above average | Average | - | + | + | + | - |
|  | M | 6 | CAS, PDis | - | Average | Average | Moderate | - | - | - | - | - |
|  | M | 6 | CAS | - | High average | Average | Average | - | + | + | + | - |
|  | M | 6;0 | CAS | - | Borderline | Severe |  | - | - | - | Features | Features |
|  | M | 6;0 | CAS, PDel | - | Average | Moderate |  | - | + | - | Features | - |
|  | M | 6;1 | CAS | - |  |  |  | - | - | - | Features | - |
|  | M | 6;2 | CAS | - | Low average |  |  | - | + | + | + | + |
|  | M | 6;2 | CAS | Features | Average | Moderate | Average | - | - | - | - | Features |
|  | F | 6;4 | CAS | + | Low average | Mild | Severe | - | + | + | + | + |
|  | M | 6;5 | CAS | - | Low average | Average | Average | - | + | + | - | - |
|  | M | 6;5 | CAS | - | Average | Average | Average | - | - | - | Features | - |
|  | M | 6;5 | CAS, PDis | - | Low average |  |  | - | + | + | + | Features |
|  | F | 6;6 | CAS, PDis | - | Low average |  |  | - | - | - | + | + |
|  | M | 6;6 | CAS, PDel | + |  |  |  | - | - | + | Features | Features |
|  | F | 6;6 | CAS | Features |  | Average | Moderate | - | + | + | + | - |
|  | F | 6;8 | CAS, DYS, PDel | - | Borderline | Severe | Mild | - | + | + | - | Features |
|  | M | 6;8 | CAS, DYS | - | Average |  |  | - | + | + | + | Features |
|  | M | 6;8 | CAS, PDel | - |  | Average | Moderate | - | - | - | - | Features |
|  | F | 6;9 | CAS | - | Average | Mild | Severe | - | - | - | + | - |
|  | F | 6;9 | CAS, PDel | - | Low average | Average | Severe | - | - | - | Features | Features |
|  | M | 6;9 | CAS | + | Average | Average | Moderate | - | - | + | - | Features |
|  | M | 7;1 | CAS | - |  | Mild | Moderate | - | - | - | - | + |
|  | M | 7;4 | CAS, PDel | - | Low average | Severe | Severe | - | - | - | + | Features |
|  | F | 7;7 | CAS, PDel | + | Borderline | Mild | Severe | - | - | + | - | - |
|  | M | 7;10 | CAS, PDel | - |  | Average | Moderate | - | + | + | - | Features |
|  | M | 8;1 | CAS, PDel | - | Average | Low average | Moderate | - | - | + | + | + |
|  | F | 8;2 | CAS, DYS | - | Low average | Severe | Moderate | - | - | + | - | + |
|  | M | 8;3 | CAS, PDel | Features | Borderline | Severe | Severe | - | + | + | Features | Features |
|  | M | 8;4 | CAS | - | Average | Average | Average | - | - | + | - | - |
|  | M | 8;6 | CAS | - | Borderline | Average | Severe | - | + | - | - | Features |
|  | M | 9;1 | CAS | - | Average |  |  | - | - | - | - | - |
|  | M | 9;3 | CAS, PDel | - | Low average | Average | Severe | - | + | - | - | - |
|  | M | 9;3 | CAS, Stutter | - | Mild | Average | Moderate | - | - | - | - | Features |
|  | M | 11;4 | CAS | Features | Mild |  |  | - | - | - | + | + |
|  | M | 11;6 | CAS | - | Average | Average | Average | - | + | + | Features |  |
|  | M | 12;2 | CAS | + | Average | Moderate | Moderate | - | - | - | - | - |

F: female; M: male; y: year: m: month; CAS: childhood apraxia of speech; PDis: Phonological disorder; PDel: Phonological delay; DYS: Dysarthria; Stutt: stuttering; +: Yes, present; -: No, not present; IQ: Full Scale Intelligence Quotient; blank: not tested; ASD: Autism spectrum disorder; ADHD: Attention deficit hyperactivity disorder
