# Supplementary material for: Childhood motor speech disorders: who to prioritise for genetic testing
Source: Eur J Hum Genet. 2026 Jan 13;34(5):639–48. doi: 10.1038/s41431-025-01993-9 (PMC13171898; doi:10.1038/s41431-025-01993-9)
Supplement: Supplementary file 3 — Supplemental Table 2a [file 41431_2025_1993_MOESM3_ESM.docx]

|  | Sex | Age y;m | Genetic diagnosis | Dysmorphology detail ails | Feeding difficulties | Hearing issues | Seizures | Other medical | Reading impairment | Spelling impairment | Education setting |
| --- | --- | --- | --- | --- | --- | --- | --- | --- | --- | --- | --- |
| 1. 1 | M | 5;5 | *ADGRL1* | Long palpebral fissures, long lashes, long columnella, slightly pointed chin | - | - | - | Adenoidectomy, allergies | NA | NA | Mainstream kinder |
| 1. 744 | M | 4;1 | *ANK2* | - | - | - | - | Tonsillectomy, adenoidectomy | NA (<5y) | NA (<5y) | Mainstream kinder |
| 1. 622 | F | 5;5 | *BPTF* | Thin body habitus, long face, small chin, low columella, hypoplastic alar nasae, short philtrum, thin upper lip, small ear lobes | - | - | - | - | + | + | Mainstream kinder |
| 1. 888 | M | 4;10 | *CACNA1A* | - | - | Grommets | Staring spells, EEG normal | - | NA (<5y) | NA (<5y) | Mainstream kinder |
| 1. 880 | M | 6;10 | *CACNA1A* | Short philtrum and rounded nasal tip | - | - | - | - | NA | NA | Mainstream prep |
| 1. 823 | M | 7;3 | *CACNA1A* | - | - | - | - | - | NA | - | Mainstream Grade 1 |
| 1. 601 | F | 3;4 | *CAMK2A* | - | - | - | - | - | NA (<5y) | NA (<5y) | Homeschool |
| 1. 420 | M | 8;0 | *CAMTA1* | Facial asymmetry | - | - | - | - | + | + | Mainstream |
| 1. 669 | M | 4;8 | *CUX1* | Broad flat nose, similar to siblings | - | - | - | - | + | + | Mainstream kinder |
| 1. 624 | M | 17 | *EBF3* | Long face, flat midface, small chin, mild ptosis | - | - | - | Plagiocephaly as infant, drooling in infancy | - | - | Mainstream |
| 1. 649 | M | 4;3 | *EHMT1* | Mildly coarse appearance | - | Grommets | - | Tonsillectomy; adenoidectomy; umbilical hernia | NA (<5y) | NA (<5y) | Mainstream childcare |
| 1. 762 | M | 6;1 | *EHMT1* | Brachycephaly, slightly flat midface, prominent arched eyebrows, neonatal teeth | - | - | - | - | NA | NA | Mainstream |
| 1. 664 | M | 6;7 | *FBXW7* | Subtle dysmorphism: flat midface, periorbital fullness, pointed chin, slight posteriorly rotated ears, down-turned corners of mouth. tall stature | - | - | - | Flat feet | + | + | Mainstream prep |
| 1. 689 | M | 4;6 | *FOXP1* | - | - | Grommets | - | Iron deficiency, high pain threshold | NA (<5y) | NA (<5y) | Telethon Speech & Hearing program 1 day/week |
| 1. 505 | F | 4;3 | *GNAI1* | Hypertelorism, fleshy facial features, almond shaped eyes, cupid's bow mouth | - | Grommets, recurrent ear infections | - | - | + | + | Mainstream childcare - pre-kindy room |
| 1. 584 | F | 4;2 | *KCND3* | - | - | Grommets | - | - | + | - | Mainstream kinder |
| 1. 694 | M | 3;7 | *KDM5C* | - | + | - | - | Cardiac septal defect resolved spontaneously, constipation, poor sleep | NA (<5y) | NA (<5y) | Mainstream kinder & long day care |
| 1. 659 | F | 5;9 | *NSD1* | Mild brachycephaly, long myopathic face, short horizontal eyebrows, blepharophimosis, flat nasal bridge, short pointed chin, hypermobile hand joints, tapering fingers, short thumbs, underdeveloped skin creases, pes planus, shortened great toes | - | - | - | Bilateral talipes treated with casting | + | + | Mainstream kinder |
| 1. 670 | F | 3;7 | *PPP2R5D* | - | - | - | - | - | NA (<5y) | NA (<5y) | Mainstream childcare |
| 1. 758 | F | 4;6 | *RAF1* | Almond shaped eyes, relatively square forehead, rounded nasal tip, prominent philtrum and thin upper lip. | + | - | - | - | NA (<5y) | NA (<5y) | Mainstream kinder |
| 1. 804 | F | 4;6 | *SCN8A* | Broad forehead, pointed chin | + | - | - | Recurrent otitis media | NA (<5y) | NA (<5y) | Mainstream kinder |
| 1. 301 | F | 10;9 | *SET* | - | - | - | - | Infantile hypotonia | NA | NA | Mainstream |
| 1. 617 | F | 3;9 | *SETBP1* | Curly hair, prominent forehead, mild hypertelorism, ptosis, sparse lateral eyebrows, retrognathia, mildly dysmorphic ears | - | - | - | Mild cleft palate, tonsillectomy | + | + | Mainstream day care |
| 1. 653 | F | 14;6 | *SETBP1* | Subtle facial dysmorphism. | + | - | - | Recurrent UTI, vesico-ureteric reflux, ureteric reimplantation | + | - | Mainstream |
| 1. 905 | M | 3;11 | *SETD1A* | Macrocephaly, broad forehead, upturned nose, cupid's bow mouth, periorbital fullness, interrupted upper ear helix, hypermobile small joints of hands, mild brachydactyly w/ mild 5th finger clinodactyly | + | - | - | Neonatal parechovirus, RSV bronchiolitis, antenatal renal abnormality (resolved) | NA (<5y) | NA (<5y) | Mainstream kinder |
| 1. 846 | M | 4;8 | *SETD2* | Slightly unusual facial features, epicanthic folds | + | Grommets | - | Adenoidectomy, tongue tie | NA (<5y) | NA (<5y) |  |
| 1. 834 | F | 5;2 | *SETD5* | Relatively large head, epicanthic folds, rounded nasal tip, flat nasal root, wide mouth, upturned upper lip | - | - | - | Hypoglycaemia with illness, recurrent abdominal pain | + | NA | Mainstream kinder |
| 1. 768 | M | 3;5 | *SLC6A1* | Periorbital fullness, full cheeks, broad nasal root, sparse and fair eyebrows, slightly upturned nose, down-turned corners of mouth | - | - | - | Eczema | NA (<5y) | NA (<5y) | Mainstream kinder |
| 1. 695 | F | 16;5 | *SLC6A8* | Narrow mouth, short philtrum, pointed chin, slightly short 5th fingers | + | - | - | - | NA | NA |  |
| 1. 673 | F | 3;6 | *SMARCA2* | Broad fleshy nasal tip, short philtrum, slightly small chin, mild pectus excavatum, short fingers with broad tips and foetal finger pads, hirsute, dark eyebrows, anterior hairline extending over temple | - | Grommets | - | Umbilical hernia, grommets, adenoidectomy. | NA (<5y) | NA (<5y) | Mainstream prep |
| 1. 586 | M | 3;9 | *SMARCA2* | Epicanthic folds, broad nose, periorbital fullness, short philtrum with prominent pillars, cupid's bow upper lip | - | Ear infections | - | - | + | + | Mainstream |
| 1. 663 | M | 7;3 | *SPTBN1* | - | + | Unilateral mild-moderate hearing impairment | - | - | NA | NA | Mainstream |
| 1. 666 | M | 13 | *SRRM2* | Mild hypotelorism, prominent eyebrows, thin upper lip, hanging columella, small jaw, bifid uvula, slightly low set ears, peripheral joint hypermobility | - | - | - | - | + | + | Mainstream |
| 1. 777 | M | 7;2 | *TAB2* |  | - | - | - | Cardiac septal defect, closed spontaneously | + | + | Mainstream prep |
| 1. 507 | M | 4;2 | *TRIM8* | Fine facial features, thin lips, broad forehead | + | - | - | - | + | + | Mainstream kinder |
| 1. 677 | M | 5;3 | 1q21.1q21.2 dup | - | - | - | - | Tonsillectomy | + | + |  |
| 1. 414 | M | 9;7 | 15q13.2q13.3 del | - | - | - | - | - | + | + | Mainstream |
| 1. 697 | M | 5;1 | 16p12.2 del | Long philtrum, thin upper lip, slightly short palpebral fissures, mild fifth finger clinodactyly | + | - | - | Inguinal hernias, epigastric hernias, poor sleep | - | + | Mainstream kinder |
| 1. 810 | M | 5;2 | 17q12 dup | - | - | - | - | - | NA | NA | Mainstream kinder |
| 1. 443 | M | 4;9 | 22q11.21 del | High forehead, wide and open mouth, broad nasal bridge, epicanthic folds, arched eyebrows with lateral spareness, prominent nasolabial folds, over-folded helix | + | Conductive hearing loss | - | Feeding difficulties, reflux; anaphylaxis to nuts, eczema, impetigo | - | - | Mainstream kinder |
| 1. 685 | M | 6;6 | 22q11.21q11.22 dup | Low set ears with fleshy ear lobes, bulbous nose, thin upper lip, webbed toes, short fingers | - | - | - | Oculomotor apraxia, poor endurance | - | - | Mainstream |
| 1. 681 | F | 4;1 | 47, XXX | Rounded nasal tip, thin upper lip, mild facial asymmetry | + | Mild conductive hearing loss in both ears, mild eustachian tube dysfunction | - | Asthma, allergies | NA (<5y) | NA (<5y) | Mainstream kinder |
| 1. 789 | M | 8;5 | 48, XXYY | Periorbital fullness, full cheeks, broad nasal root, sparse and fair eyebrows, slightly upturned nose, down-turned corners of mouth | - | - | - | - | + | + | Mainstream |
| 1. 794 | M | 6 | 48, XXYY | - | - | - | - | Asthma, allergies, tall stature, anxiety | - | - | Mainstream kinder |

F: female; M: male; y: year: m: month; +: present; -: not present; Grommets also known as middle ear ventilation tympanostomies; NA (<5 y): indicates children were too young to determine literacy performance (typically assessed > 5 years after school entry); prep: preparatory (first year of school; also known as Foundation year).
