# Supplementary material for: Childhood motor speech disorders: who to prioritise for genetic testing
Source: Eur J Hum Genet. 2026 Jan 13;34(5):639–48. doi: 10.1038/s41431-025-01993-9 (PMC13171898; doi:10.1038/s41431-025-01993-9)
Supplement: Supplementary file 4 — Supplemental Table 2b [file 41431_2025_1993_MOESM4_ESM.docx]

|  | Sex | Age y;m | Dysmorphology detail | Feeding difficulties | Hearing issues | Seizures | Other medical | Reading impairment | Spelling impairment | Education setting |
| --- | --- | --- | --- | --- | --- | --- | --- | --- | --- | --- |
|  | M | 2;7 |  | - | - | - |  | NA (<5y) | NA (<5y) | Maintstream play group |
|  | M | 3;1 |  | - | - | - |  | NA (<5y) | NA (<5y) | Maintstream kinder |
|  | M | 3;1 |  | - | - | - |  | NA (<5y) | NA (<5y) | Maintstream daycare |
|  | M | 3;2 |  | + | - | - |  | NA (<5y) | NA (<5y) | Maintstream childcare |
|  | M | 3;2 |  | - | - | - |  | NA (<5y) | NA (<5y) | Maintstream daycare 2/days a week |
|  | M | 3;2 | Cupids bow mouth, slightly high nasal root slightly deep-set eyes | - | - | - | Congenital talipes equinovarus, enlarged tonsils | NA (<5y) | NA (<5y) | Maintstream daycare |
|  | M | 3;3 |  | + | - | - |  | NA (<5y) | NA (<5y) | Maintstream playgroup |
|  | F | 3;4 | Thin body build, thin fair hair | - | - | - |  | NA (<5y) | NA (<5y) | Mainstream daycare |
|  | F | 3;4 |  | + | - | - | Inguinal hernia repair, esotropia | NA (<5y) | NA (<5y) | Mainstream daycare |
|  | M | 3;4 | Triangular face, flat occiput | + | Grommets | - | Gastroesophageal reflux, plagiocephaly, egg allergy | NA (<5y) | NA (<5y) | Mainstream daycare |
|  | M | 3;5 |  | - | - | - |  | NA (<5y) | NA (<5y) | Mainstream daycare |
|  | F | 3;5 | Fine features, small chin, square forehead, slightly short fifth fingers with clinodactyly | - | - | - |  | NA (<5y) | NA (<5y) | Mainstream kinder |
|  | M | 3;6 |  | - | - | - | Duane retraction syndrome. Brain MRI shows stenotic left internal auditory canal with thick modiolus and absent left cochlear nerve, with some flattening of pons on R side (moebius-like). | NA (<5y) | NA (<5y) | Mainstream kinder |
|  | M | 3;6 |  | + | Grommets | - | Adenoidectomy, tongue tie release |  |  | Mainstream childcare |
|  | M | 3;6 | Broad forehead, fair eyebrows, slightly upslanting palpebral fissures | - | Grommets | - | Tonsillectomy, adenoidectomy | NA (<5y) | NA (<5y) | Mainstream kinder |
|  | M | 3;7 |  | - | - | - | Hydrocele | NA (<5y) | NA (<5y) | Mainstream kinder |
|  | M | 3;7 |  | + | - | - |  | NA (<5y) | NA (<5y) | Mainstream daycare |
|  | M | 3;7 |  | - | Grommets | - | Adenoidectomy | NA (<5y) | NA (<5y) | Mainstream daycare |
|  | F | 3;8 | Round face, full cheeks | - | Grommets | - |  | NA (<5y) | NA (<5y) | Mainstream daycare |
|  | M | 3;8 |  | + | Grommets | - | Asthma | NA (<5y) | NA (<5y) | Mainstream kinder |
|  | M | 3;11 | Unusual ear helices | - | Grommets | - |  | NA (<5y) | NA (<5y) | Mainstream childcare |
|  | M | 3;11 |  | - | - | - |  | NA (<5y) | NA (<5y) | Mainstream kinder |
|  | M | 4 |  | + | - | - |  | - | - | Mainstream kinder |
|  | F | 4 | Found face, square forehead, mild hypertelorism | - | - | - | Constipation, low iron, recurrent abdominal pain, UTIs | NA (<5y) | NA (<5y) | 1 day 3-year-old kinder, 2 days childcare |
|  | M | 4 |  | - | - | - | Urticaria, food allergies | NA (<5y) | NA (<5y) | Mainstream daycare |
|  | M | 4;0 |  | - | Grommets | - | Recurrent ear infections, umbilical hernia, hydrocoele, drooling, iron deficiency anaemia | NA (<5y) | NA (<5y) | Mainstream kinder |
|  | M | 4;0 | Mild hypotelorism, deep set eyes | - | - | 2x febrile seizures | Tongue and lip tie release | NA (<5y) | NA (<5y) | Mainstream kinder |
|  | M | 4;1 |  | - | - | - |  | NA (<5y) | NA (<5y) | Play group 1.5 hours 1 day week |
|  | M | 4;2 |  | - | - | - |  | NA (<5y) | NA (<5y) | No |
|  | M | 4;2 |  | - | - | - |  | NA (<5y) | NA (<5y) | Mainstream kinder |
|  | F | 4;3 |  | + | - | - | Squint surgery | NA (<5y) | NA (<5y) | Mainstream daycare |
|  | M | 4;3 |  | - | - | - |  | - |  | Mainstream daycare |
|  | F | 4;4 |  |  | Eustachian tube dysfunction | - |  | + | + | Yarragon Early Learning Centre |
|  | M | 4;4 |  | - | - | - |  | NA (<5y) | NA (<5y) | Mainstream kinder |
|  | F | 4;4 |  | - | - | - | Hypotonia, strabismus, allergies | NA (<5y) | NA (<5y) | Mainstream kinder |
|  | M | 4;4 |  | - | - | - | Tall stature, joint hypermobility, tonsillectomy, adenoidectomy | NA (<5y) | NA (<5y) | Mainstream kinder |
|  | M | 4;4 |  | + | - | - |  | NA (<5y) | NA (<5y) | Mainstream kinder |
|  | M | 4;4 | Fifth finger clinodactyly | - | - | - | Umbilical hernia, eczema | NA (<5y) | NA (<5y) | Mainstream kinder |
|  | M | 4;5 |  | - | Grommets | - | Tonsillectomy and adenoidectomy | NA (<5y) | NA (<5y) | Mainstream kinder |
|  | M | 4;6 |  | + | - | - | Ventricular septal defect | NA (<5y) | NA (<5y) | Mainstream kinder |
|  | F | 4;7 | Oval shaped face, epicanthic folds, mildly arched eyebrows, broad/flat nose | - | - | - |  | NA (<5y) | NA (<5y) | Mainstream daycare |
|  | M | 4;8 |  | - | Grommets | - | Asthma, poor sleep, constipation | + | + | Mainstream kinder |
|  | F | 4;8 |  | - | - | - | Keratosis pilaris | NA (<5y) | NA (<5y) | Mainstream childcare |
|  | M | 4;8 |  | - | - | - |  | + | + | Mainstream kinder |
|  | M | 4;8 |  | - | - | - |  |  |  | Mainstream kinder |
|  | F | 4;9 | Round face, fleshy facial features, full lips, upturned nose | - | - | - |  | - | + | Mainstream kinder |
|  | M | 4;9 | Slightly fleshy & uplifted ear lobes, short palpebral fissures, relatively flat nose, short philtrum | - | - | - |  | + | + | Mainstream kinder |
|  | M | 4;9 |  | - | - | - |  |  |  | Mainstream kinder |
|  | M | 4;9 |  | - | - | - | Tonsilectomy, adnoidectomy | NA (<5y) | NA (<5y) | Mainstream kinder |
|  | F | 4;9 |  | - | - | - | Peanut allergy, asthma, eczema | NA (<5y) | NA (<5y) | Mainstream kinder |
|  | M | 4;10 |  | - | - | - |  | - | + |  |
|  | F | 5 | Mild brachydactyly of hands, fifth finger clinodactyly | - | - | - |  | + | + | Mainstream kinder |
|  | F | 5 |  | - | - | - |  |  |  | Mainstream preschool |
|  | M | 5;0 |  | - | - | - | Overlapping toes |  |  | Mainstream daycare |
|  | F | 5;1 |  | - | - | - |  | - | - | Mainstream kinder |
|  | F | 5;1 |  | - | Sensorineural hearing loss | - |  |  |  | Mainstream kinder |
|  | M | 5;1 |  | - | - | - | Oculomotor apraxia |  |  | Mainstream kinder |
|  | M | 5;2 | Fleshy facial features & hands | + | - | - | Obesity | + | + | Mainstream kinder |
|  | F | 5;2 | Fine facial features, slightly short palpebral fissures | - | - | - |  |  |  | Mainstream kinder |
|  | F | 5;3 |  | - | - | - | Tonsillectomy, adenoidectomy | - | - | Mainstream kinder |
|  | M | 5;4 |  | - | - | - | Adenoidectomy |  |  | Mainstream kinder |
|  | M | 5;4 |  | + | Grommets | - | Poor sleep, obstructive sleep apnoea, adenoidectomy, turbinectomy, gastroesophageal reflux, chronic constipation, joint hypermobility |  |  |  |
|  | M | 5;5 | Relatively long nose, smooth philtrum, thin upper lip pointed chin, slightly stooped posture with rounded shoulders | + | - | - | Adenoidectomy, tonsilectomy, constipation |  |  | Mainstream kinder |
|  | M | 5;5 |  | - | - | - |  |  |  | Mainstream daycare |
|  | M | 5;6 |  | + | Mild conductive hearing loss in L) ear | - |  | - | - | Mainstream kinder |
|  | M | 5;6 | Prominent forehead, periorbital fullness with superior epicanthic folds, slightly small ears | + | - | - | Asthma, tonsillectomy, adenoidectomy, recurrent virus infections | - | - | Mainstream kinder |
|  | F | 5;6 | Overbite/malocclusion | - | - | - |  |  |  | Mainstream kinder |
|  | M | 5;6 |  | + | - | - | Tonsillectomy, tongue tie |  |  | Mainstream kinder |
|  | M | 5;7 |  | - | - | - |  |  |  | Mainstream kinder |
|  | M | 5;9 |  | - | - | - | Enlarged tonsils and adenoids, allergies | + | + | Individualised learning plan in mainstream prep |
|  | M | 5;9 | Mild plagiocephaly, brachycephaly, bright blue eyes, thin upper lip, rounded nasal tip & mildly hypoplastic alar nasae | - | Grommets | - | Adenoidectomy, eardrum perforation | - | - | Mainstream prep |
|  | M | 5;9 |  | - | - | - |  | + | + | Mainstream kinder |
|  | M | 5;10 |  | - | - | - | Recurrent otitis media, asthma |  |  | Mainstream prep |
|  | M | 5;11 |  | - | - | - |  |  |  | Mainstream prep |
|  | M | 6;0 |  | - | - | - |  | + | - | Mainstream prep |
|  | M | 6;0 |  | - | - | - | Type 1 diabetes, eczema, asthma | - | + | Mainstream prep |
|  | M | 6;0 |  | - | - | - | Recurrent otitis media | + | + | Mainstream Prep |
|  | M | 6;0 |  | - | - | - |  |  |  | Mainstream kinder |
|  | M | 6;1 |  | - | - | - |  |  |  | Mainstream kinder |
|  | M | 6;2 |  | - | - | - | Poor sleep |  |  | Mainstream kinder |
|  | M | 6;2 | Thin body build, small chin/retrognathia | + | - | - | Short stature, recurrent otitis media and viral infections |  |  | Mainstream prep |
|  | F | 6;4 |  | + | Grommets | - | Tonsillectomy-and adenoidectomy, grommets, long sighted | + | + | Mainstream prep |
|  | M | 6;5 |  | + | - | - |  | - | + | Mainstream prep |
|  | M | 6;5 |  | - | - | - | Chronic renal impairment | + | - | Mainstream kinder |
|  | M | 6;5 |  | - | Grommets | - | Adenoidectomy | - | - | Mainstream prep |
|  | F | 6;6 |  | - | - | - |  | + | + | Mainstream |
|  | M | 6;6 |  | - | - | - | Asthma |  |  | Mainstream |
|  | F | 6;6 | Slightly down slanting palpebral fissures, prominent nasolabial folds | - | - | - | Right brachial sinus repaired at 18 months, recurrent headache, UTI, htypotonia |  |  | Mainstream |
|  | F | 6;8 |  | - | + | - | Tonsillectomy | + | + | Mainstream prep |
|  | M | 6;8 |  | - | - | - | Neonatal hip dysplasia, bicuspid aortic valve |  |  | Mainstream |
|  | M | 6;8 |  | - | - | - |  |  |  | Mainstream |
|  | F | 6;9 |  | + | Grommets | - | Adenoidectomy. | - | - | Mainstream |
|  | F | 6;9 |  | - | - | - | Tonsillectomy and adenoidectomy | + | + | Mainstream prep |
|  | M | 6;9 | Cupid's bow upper lip, down turned corners of mouth, slightly flat midface, horizontal eyebrows, high forehead, crowded facial features | - | Grommets | - | Sleep-disordered breathing, recurrent otitis media, adenoictemy, septic arthritis right knee, mild obesity | + | - | Mainstream |
|  | M | 7;1 |  | - | - | - |  | + |  | Mainstream |
|  | M | 7;4 |  | - | - | - | Recurrent ear infections, adenoidectomy | + | + | Mainstream |
|  | F | 7;7 | Short palpebral fissures, almond shaped eyes, wide mouth, high forehead | - | - | - | Allergies | + | + | Mainstream |
|  | M | 7;10 |  | - | - | - | Eczema, gastroesophageal reflux | + | + | Mainstream prep |
|  | M | 8;1 |  | - | Grommets | - | Urinary incontinence following fractured femur, spinal cord tethering, tremor, tonsillectomy, adenoidectomy | + | - | Mainstream |
|  | F | 8;2 |  | - | - | - |  | + | + | Mainstream (repeated yr 1) |
|  | M | 8;3 | Prominent front upper teeth, prominent left ear, slightly broad thumbs | - | Grommets | - | Adenotonsillectomy | + |  | Mainstream |
|  | M | 8;4 |  | - | - | - | Eczema, joint hypermobility | - | + | Mainstream |
|  | M | 8;6 |  | - | - | + |  |  |  | Mainstream |
|  | M | 9;1 |  | - | - | - |  | + |  | Mainstream |
|  | M | 9;3 |  | - | - | - |  | + | + | Mainstream |
|  | M | 9;3 |  | - | - | - | Tonsillectomy | + | + | Mainstream |
|  | M | 11;4 | Right pre-helical ear pit, metatarsus adductus with broad great toes, plagiocephaly | - | Grommets | - | Reduced muscle tone with intact reflexes. | + | + | Specialist |
|  | M | 11;6 |  | - | - | - |  | + | + | Mainstream |
|  | M | 12;2 | Mild hypotelorism, slightly prominent nose, short philtrum, small mouth, thin upper lip consistent with previous cleft repair, increased small joint hypermobility of hands | - | - | - | Surgeries for cleft palate | - | - | Mainstream |

F: F: female; M: male; y: year: m: month; + : present; -: not present; Grommets also known as middle ear ventilation tympanostomies; NA (<5 y): indicates children were too young to determine literacy performance (typically assessed > 5 years after school entry); prep: preparatory (first year of school; also known as Foundation year).
