# Supplementary material for: Childhood motor speech disorders: who to prioritise for genetic testing
Source: Eur J Hum Genet. 2026 Jan 13;34(5):639–48. doi: 10.1038/s41431-025-01993-9 (PMC13171898; doi:10.1038/s41431-025-01993-9)
Supplement: Supplementary file 5 — Supplemental Table 3 [file 41431_2025_1993_MOESM5_ESM.docx]

3a. Monogenic variants of unknown significance in participants with genetic diagnoses

| ID | Gene | Chr:Pos  (GRCh38/hg38) | Variant (GRCh38/hg38) | Inheritance | In silico predictions^a^ | gnomAD allele count (v4.1) | ACMG class | Type |
| --- | --- | --- | --- | --- | --- | --- | --- | --- |
| 8 | *FOXP2* | chr7:114658217 | c.1418G>A; p.(Gly473Glu) | *De novo* | REVEL = Uncertain(0.57)  AlphaMissense = Del(0.905)  SIFT = Uncertain(0.002)  MT = Del(1) | 0 | 3 | Missense |
| 5 | *TRRAP* | chr7:98903517 | c.1036C>T; p.(Gln346*) | Maternal | CADD = Del(41)  MT = Del(1) | 0 | 3 | Nonsense |

3b. Monogenic variants of unknown significance in participants without genetic diagnoses

| ID | Gene | Chr:Pos  (GRCh38/hg38) | Variant (GRCh38/hg38) | Inheritance | In silico predictions^a^ | gnomAD allele count (v4.1) | ACMG class | Type |
| --- | --- | --- | --- | --- | --- | --- | --- | --- |
| 71 | *AP1G1* | chr16:71769701 | c.566-2A>G | Paternal | SpliceAI = Splice-Altering(1)  Ada = Del(1)  RF = Del(0.93) | 0 | 3 | Canonical splice site |
| 98 | *BCORL1* | chrX:130014790 | c.2018G>T; p.(Gly673Val) | Maternal | REVEL = Benign(0.15)  AlphaMissense = Benign(0.122)  SIFT = Uncertain(0.003)  MT = Del(0.75) | 43 | 3 | Missense |
| 134 | *BRPF1* | chr3:9743628 | c.2362G>A; p.(Val788Met) | *De novo* | REVEL = Benign(0.03)  AlphaMissense = Benign(0.098)  SIFT = Uncertain(0.0056)  MT = Del(0.75) | 0 | 3 | Missense |
| 105 | *CRY1* | chr12:107092946 | c.16G>A; p.(Val6Met) | *De novo* | REVEL = Uncertain(0.29)  AlphaMissense = Uncertain(0.622)  SIFT = Uncertain(0.001)  MT = Del(1) | 2 | 3 | Missense |
| 97 | *DPF2* | chr11:65345761 | c.733C>G; p.(Gln245Glu) | *De novo* | REVEL = Benign(0.27)  AlphaMissense = Benign(0.071)  SIFT = Benign(1)  MT = Del(1) | 1 | 3 | Missense |
| 47 | *FGF13* | chrX:139204139 | c.-32C>G | Maternal | NA | 45 | 3 | Noncoding |
| 49 | *IQSEC2* | chrX:53243451 | c.2770A>G; p.(Ile924Val) | Maternal | REVEL = Benign(0.24)  AlphaMissense = Del(0.93)  SIFT = Uncertain(0.019)  MT = Del(1) | 7 | 3 | Missense |
| 145 | *USP9X* | chrX:41197449 | c.4319T>C; p.(Phe1440Ser) | Maternal | REVEL = Uncertain(0.63)  AlphaMissense = Del(0.987)  SIFT = Uncertain(0.002)  MT = Del(1) | 0 | 3 | Missense |
| 121 | *USP9X* | chrX:41215995 | c.5428A>G; p.(Ile1810Val) | Maternal | REVEL = Benign(0.16)  AlphaMissense = Benign(0.165)  SIFT = Benign(0.175)  MT = Del(1) | 15 | 3 | Missense |

Abbreviations: ACMG = American College of Medical Genetics; Chr = chromosome; Del = deleterious; Pos = position

^a^In silico interpretation:

Ada = AdaBoost; predicts potential for altering splicing within splicing consensus regions using AdaBoost algorithm; range 0-1 (0 = benign, 1 = disease causing)

AlphaMissense = predicts missense variant pathogenicity by combing structural context and evolutionary conservation; range 0-1 (0 = benign, 1 = deleterious)

CADD = Combined Annotation Dependent Depletion; integrates multiple annotations into one metric by contrasting variants that survived natural selection with simulated mutations; 0:Benign, >10:Deleterious

MT = MutationTaster; employs a Bayes classifier to predict the disease potential of an alteration; range 0-1 (0 = benign, 1 = deleterious)

REVEL = Rare Exome Variant Ensemble Learner; ensemble method, predicting missense pathogenicity based on combination of 13 in silico tools; range 0-1 (0 = benign, 1 = deleterious)

RF = Random forest; predicts potential for altering splicing within splicing consensus regions using random forest algorithm; range 0-1 (0 = benign, 1 = deleterious)

SIFT = Sorting Intolerant From Tolerant; predicts whether amino acid substitution affects protein function based on degree of conservation; (0 = benign, 1 = deleterious)

SpliceAI = employs neural networks to predict if splicing event occurs; range 0-1 (0=benign, 1= splice altering)

3c. Copy number variants (CNVs) of unknown significance in participants without genetic diagnoses

| ID | Genetic finding | Molecular karyotype (GRCh38/hg38) | Inheritance |
| --- | --- | --- | --- |
| 115 | 2q37.2q37.3 del | 2q37.2-37.3(chr2:235014182-237461504)x1 | Maternal |
| 85 | 3p14.2 del | 3p14.2(chr3:62526259-62825083)x1 | *De novo* |
| 113 | 7p22.3 del | 7p22.3(ch7:1436742-1798288)x1 | Maternal |
| 81 | 14q12 del | 14q12(chr14:29558905-31072968)x1 | *De novo* |
