# Supplementary material for: Childhood motor speech disorders: who to prioritise for genetic testing
Source: Eur J Hum Genet. 2026 Jan 13;34(5):639–48. doi: 10.1038/s41431-025-01993-9 (PMC13171898; doi:10.1038/s41431-025-01993-9)
Supplement: Supplementary file 6 — Supplemental Table 4 [file 41431_2025_1993_MOESM6_ESM.docx]

Supplemental Table 4. Evidence for speech motor candidate genes also associated with other neurodevelopmental phentoypes in PanelApp Australia (green evidence)

|  | PanelApp panel | | | |
| --- | --- | --- | --- | --- |
| Speech motor genes | Intellectual disability | Epilepsy | Autism | Cerebral  palsy^VV^ |
| *ADGRL1* | + | - | - | - |
| *ANK2* | + | + | + | - |
| *BPTF* | + | + | - | - |
| *CACNA1A* | + | + | - | + |
| *CAMK2A* | + | + | - | - |
| *CAMTA1* | + | - | - | + |
| *CUX1* | + | - | - | - |
| *EBF3* | + | - | - | - |
| *EHMT1* | + | + | - | - |
| *FBXW7* | + | - | - | - |
| *FOXP1* | + | + | + | - |
| *GNAI1* | + | + | - | - |
| *KCND3* | + | + | - | - |
| *KDM5C* | + | + | - | + |
| *NSD1* | + | + | + | - |
| *PPP2R5D* | + | + | + | - |
| *RAF1* | + | - | - | - |
| *SCN8A* | + | + | - | + |
| *SET* | + | - | - | - |
| *SETBP1* | + | + | - | - |
| *SETD1A* | + | + | - | - |
| *SETD2* | + | - | - | - |
| *SETD5* | + | + | + | - |
| *SLC6A1* | + | + | + | - |
| *SLC6A8* | + | + | - | - |
| *SMARCA2* | + | + | + | - |
| *SPTBN1* | + | + | - | - |
| *SRRM2* | + | - | - | - |
| *TAB2* | + | - | - | - |
| *TRIM8* | + | + | - | - |

| ^VV^ Far fewer studies of CP - a number our current genes are on the red or amber lists for CP  NB: ADHD not included here as not tested clinically, so no panel App available |
| --- |
