# Supplementary material for: Childhood motor speech disorders: who to prioritise for genetic testing
Source: Eur J Hum Genet. 2026 Jan 13;34(5):639–48. doi: 10.1038/s41431-025-01993-9 (PMC13171898; doi:10.1038/s41431-025-01993-9)
Supplement: Supplementary file 7 — Supplemental Table 5 [file 41431_2025_1993_MOESM7_ESM.docx]

|  | Supplemental Table 5a. Phenotype of cohort overall (n=153) and stratified by presence (n=44) or absence (n=109) of genetic diagnosis | | | | | | |
| --- | --- | --- | --- | --- | --- | --- | --- |
|  | | **Overall** | | Genetic diagnosis | | | |
|  | |  |  | **No** | | **Yes** | |
|  | | Missing n (%) | n=153^a^ | Missing n (%) | n=109^a^ | Missing n (%) | n=44^a^ |
| Sex: female | | 0 (0) | 42 (27.5) | 0 (0) | 27 (24.8) | 0 (0) | 15 (34.1) |
| Age: months - median [IQR] | | 0 (0) | 61 [50, 77] | 0 (0) | 61 [49, 76] | 0 (0) | 61.5 [50, 83] |
| Speech diagnosis | | 1 (0.7) |  | 1 (0.9) |  | 0 (0) |  |
|  | CAS only |  | 77 (50.3) |  | 59 (54.1) |  | 18 (40.9) |
|  | CAS and other(s) |  | 74 (48.4) |  | 50 (45.9) |  | 24 (54.5) |
|  | Dysarthria |  | 2 (1.3) |  | 0 (0) |  | 2 (4.5) |
| Gross motor impairment | | 0 (0) | 71 (46.4) | 0 (0) | 41 (37.6) | 0 (0) | 30 (68.2) |
| Fine motor impairment | | 0 (0) | 73 (47.7) | 0 (0) | 44 (40.4) | 0 (0) | 29 (65.9) |
| Delayed walking | | 0 (0) | 30 (19.6) | 0 (0) | 7 (6.4) | 0 (0) | 23 (52.3) |
| Vision impairment | | 0 (0) | 22 (14.4) | 0 (0) | 16 (14.7) | 0 (0) | 6 (13.6) |
| Hearing issues | | 0 (0) |  | 0 (0) |  | 0 (0) |  |
|  | Mild hearing impairment |  | 8 (5.2) |  | 4 (3.7) |  | 4 (9.1) |
|  | Grommets |  | 27 (17.6) |  | 19 (17.4) |  | 8 (18.2) |
|  | Absent |  | 118 (77.1) |  | 86 (78.9) |  | 32 (72.7) |
| Seizures | | 0 (0) | 3 (2.0) | 0 (0) | 2 (1.8) | 0 (0) | 1 (2.3) |
| ASD diagnosis | | 0 (0) |  | 0 (0) |  | 0 (0) |  |
|  | Present |  | 24 (15.7) |  | 20 (18.3) |  | 4 (9.1) |
|  | Autistic features |  | 41 (26.8) |  | 34 (31.2) |  | 7 (15.9) |
|  | Absent |  | 88 (57.5) |  | 55 (50.5) |  | 33 (75.0) |
| ADHD diagnosis | | 0 (0) |  | 0 (0) |  | 0 (0) |  |
|  | Present |  | 17 (11.1) |  | 15 (13.8) |  | 2 (4.5) |
|  | ADHD features |  | 28 (18.3) |  | 25 (22.9) |  | 3 (6.8) |
|  | Absent |  | 108 (70.6) |  | 69 (63.3) |  | 39 (88.6) |
| Dysmorphism | | 0 (0) |  | 0 (0) |  | 0 (0) |  |
|  | Present |  | 26 (17.0) |  | 6 (5.5) |  | 20 (45.5) |
|  | Dysmorphic features |  | 31 (20.3) |  | 22 (20.2) |  | 9 (20.5) |
|  | Absent |  | 96 (62.7) |  | 81 (74.3) |  | 15 (34.1) |
| Macrocephaly | | 1 (0.7) | 6 (3.9) | 1 (0.9) | 4 (3.7) | 0 (0) | 2 (4.5) |
| Early feeding difficulties | | 2 (1.3) | 33 (21.9) | 1 (0.9) | 21 (19.4) | 1 (2.3) | 12 (27.9) |
| Receptive language | | 44 (28.8) |  | 34 (31.2) |  | 10 (22.7) |  |
|  | Above average |  | 5 (4.59) |  | 5 (6.67) |  | 0 (0.0) |
|  | Average |  | 58 (53.21) |  | 49 (65.33) |  | 9 (26.47) |
|  | Mildly impaired |  | 20 (18.35) |  | 10 (13.33) |  | 10 (29.41) |
|  | Moderately impaired |  | 12 (11.01) |  | 5 (6.67) |  | 7 (20.59) |
|  | Severely impaired |  | 14 (12.84) |  | 6 (8.00) |  | 8 (23.53) |
| Expressive language | | 28 (18.3) |  | 22 (20.2) |  | 6 (13.6) |  |
|  | Average |  | 30 (24.00) |  | 26 (29.89) |  | 4 (10.53) |
|  | Mildly impaired |  | 13 (10.4) |  | 7 (8.05) |  | 6 (15.79) |
|  | Moderately impaired |  | 16 (12.8) |  | 12 (13.79) |  | 4 (10.53) |
|  | Severely impaired |  | 41 (32.8) |  | 24 (27.59) |  | 17 (44.74) |
|  | Minimally verbal |  | 7 (5.6) |  | 5 (5.75) |  | 2 (5.26) |
|  | Not assessed |  | 18 (14.4) |  | 13 (14.94) |  | 5 (13.16) |
| FSIQ | | 40 (26.1) |  | 30 (27.5) |  | 10 (22.7) |  |
|  | Very superior |  | 1 (0.88) |  | 1 (1.27) |  | 0 (0.0) |
|  | Superior |  | 2 (1.77) |  | 2 (2.53) |  | 0 (0.0) |
|  | High average |  | 11 (9.73) |  | 9 (11.39) |  | 2 (5.9) |
|  | Average |  | 37 (32.74) |  | 31 (39.24) |  | 6 (17.6) |
|  | Low average |  | 31 (27.43) |  | 24 (30.38) |  | 7 (20.6) |
|  | Borderline ID |  | 20 (17.70) |  | 9 (11.39) |  | 11 (32.4) |
|  | Mild ID |  | 11 (9.73) |  | 3 (3.80) |  | 8 (23.5) |

^a^Reporting n (%) unless stated otherwise

CAS: Childhood apraxia of speech; ASD: Autism spectrum disorder; ADHD: Attention deficit hyperactivity disorder; FSIQ: Full scale intelligence quotient; ID: Intellectual disability.

Supplemental Table 5b: Estimated odds ratios of genetic findings for different phenotypes

|  | Odds ratio (OR) | 95% CI |
| --- | --- | --- |
| Gross motor impairment | 3.55 | (1.72, 7.65) |
| Fine motor impairment | 2.86 | (1.39, 6.06) |
| Delayed walking | 15.96 | (6.34, 44.86) |
| Vision impairment | 0.92 | (0.31, 2.42) |
| Hearing issues | 1.4 | (0.61, 3.11) |
| Seizures | 1.24 | (0.06, 13.32) |
| ASD (present) | 0.34 | (0.15, 0.72) |
| ADHD (present) | 0.22 | (0.07, 0.56) |
| Dysmorphism (present/features) | 5.59 | (2.66, 12.19) |
| Macrocephaly | 1.24 | (0.17, 6.59) |
| Early feeding difficulties | 1.6 | (0.69, 3.61) |
| Receptive language: impaired | 7.14 | (2.96, 18.62) |
| Expressive language: impaired | 3.62 | (1.28, 13.04) |
| FSIQ: impaired | 7.07 | (2.89, 18.18) |
